# Trachoma-associated morbidity and mortality in Brazil: an ecological study focusing on hospitalization and mortality data, 2000−2022

**DOI:** 10.1590/0037-8682-0158-2024

**Published:** 2024-09-02

**Authors:** Adjoane Maurício Silva Maciel, Anderson Fuentes Ferreira, Nádia Maria Girão Saraiva de Almeida, Manuella Maurício Silva Maciel, Taynara Lais Silva, Mirele Coelho Araújo, Roberto da Justa Pires, Alberto Novaes Ramos

**Affiliations:** 1 Universidade Federal do Ceará, Faculdade de Medicina, Programa de Pós-Graduação em Saúde Pública, Fortaleza, CE, Brasil.; 2 Secretaria Municipal de Saúde, Russas, CE, Brasil.; 3 Universidade Estadual do Ceará, Mestrado Profissional em Saúde da Criança e do Adolescente, Fortaleza, CE, Brasil.; 4 Universidade Federal do Ceará, Instituto de Cultura e Arte, Fortaleza, CE, Brasil.; 5 Universidade Federal do Ceará, Faculdade de Medicina, Departamento de Saúde Comunitária, Fortaleza, CE, Brasil.

**Keywords:** Trachoma, Hospitalization, Mortality, Ecological study, Brazil

## Abstract

**Background:**

Trachoma is the leading infectious cause of blindness worldwide. It is a neglected tropical disease caused by *Chlamydia trachomatis*. The objective of this study was to analyze the trachoma-associated morbidity and mortality in Brazil from 2000 to 2022. This ecological time-series study was based on secondary data on trachoma obtained from hospital admissions (trachoma as the primary or secondary cause) and death certificates (trachoma as the underlying or associated cause).

**Methods:**

We calculated the sex- and age-standardized rates of hospital admissions and trachoma-specific mortality according to sociodemographic variables and analyzed the spatial distribution.

**Results:**

We identified 141/263,292,807 hospital admissions (primary cause: 83.0%) and 126/27,596,830 death certificates (associated cause: 91.3%) related to trachoma. Trachoma-related sequelae were reported in 8.5% of hospital admissions and 6.3% of death certificates. Trachoma was more common in males (hospital admissions and death certificates), people aged ≥70 years (hospital admissions and death certificates), those with brown skin (hospital admissions and death certificates), and those living in the North (hospital admissions) and Northeast (death certificates) regions of Brazil.

**Conclusions:**

Despite the relatively low rates of trachoma morbidity in Brazil, the associated mortality rates are of concern. The heterogeneous patterns of occurrence in the country in terms of population and territory reinforce the need to evaluate and monitor the available data, despite the low prevalence, in order to achieve and maintain the elimination targets in Brazil in the future.

## INTRODUCTION

Trachoma is the primary infectious cause of blindness globally^1^. It is a neglected tropical disease (NTD)^2^ caused by *Chlamydia trachomatis* ^1^ *.* Trachoma has strong social determinants related to poverty and other vulnerable conditions^3^. Accounting for 1.4% of global blindness cases, an estimated 115.7 million individuals resided in the endemic regions of trachoma in 2023, with 1.5 million experiencing sequelae from the disease across 40 countries^1^.

To evaluate the endemicity of the disease and declare it a public health concern, the World Health Organization (WHO) relies on prevalence indicators such as trachomatous inflammation−follicular in children aged 1-9 years, prevalence of trachomatous trichiasis (TT) in individuals aged ≥15 years who are “*unknown to the health system*,” and evidence of the health system’s capacity to identify and manage incident TT cases^1^.

During the initial phase of the national trachoma survey in non-indigenous areas from 2018 to 2019 in Brazil, the country’s overall prevalence was highlighted at a technical level to eliminate the disease^4^.

Despite implementing a national survey in Brazil that aimed to assess the endemicity of trachoma at the population level, the Trachoma Surveillance and Control Program (in Portuguese: *Programa de Vigilância e Controle do Tracoma* [PVCT]) in the country is structured around evidence gathered from surveys of school children. This involves finding active cases, treatment and treatment monitoring, health promotion, prevention, control, and disease surveillance^5^. The effectiveness of the PVCT’s actions is evaluated by monitoring the percentage of the population receiving treatment, the eligible population undergoing TT surgery, and the positivity rate^5^. The positivity rate, indicating the proportion of individuals testing positive among those screened, is commonly used to assess survey results, with thresholds set as follows: <5%, low positivity; 5-10%, medium positivity; and ≥10%, high positivity^5^.

Despite the issuance of the latest Decree No. 217 on March 1, 2023^6^, trachoma remains under national surveillance in Brazil, with elimination efforts centered on reporting aggregated data via the “*Trachoma Survey Bulletin*” in the Brazil Information System for Notifiable Diseases (in Portuguese: *Sistema de Informação de Agravos de Notificação* [SINAN])^5^. However, routine analyses currently do not include the hospital morbidity and mortality data^7^ ^,^ ^8^.

The limitations of data collection in low-endemicity contexts and characterization of only positive trachoma cases in the SINAN without recording the demographic and clinical data of those examined for the disease and their contacts hinder a comprehensive analysis of the epidemiological situation^9^. Furthermore, the low sensitivity of the healthcare and surveillance network of the Unified Health System (in Portuguese: *Sistema Único de Saúde* [SUS]), along with limited prioritization, is evident from the scarcity of scientific publications on trachoma in Brazil^10^.

Although trachoma is not directly linked to mortality, analyzing the hospital and general morbidity and mortality data highlights the vulnerabilities associated with the disease, particularly regarding access to SUS care. This study examined the trachoma-related morbidity and mortality in Brazil from 2000 to 2022 using data from the Hospital Information System (in Portuguese: *Sistema de Informações Hospitalares* [SIH]) and the Mortality Information System (in Portuguese: *Sistema de Informação de Mortalidade* [SIM]) of the SUS. This study provides a comprehensive understanding of the attention, surveillance, and control measures required for trachoma.

## METHODS

This was an ecological time-series analysis based on secondary data of hospital admissions (HA) and death certificates (DC) associated with trachoma in the different regions and states of Brazil from 2000 to 2022.

**FIGURE 1:**
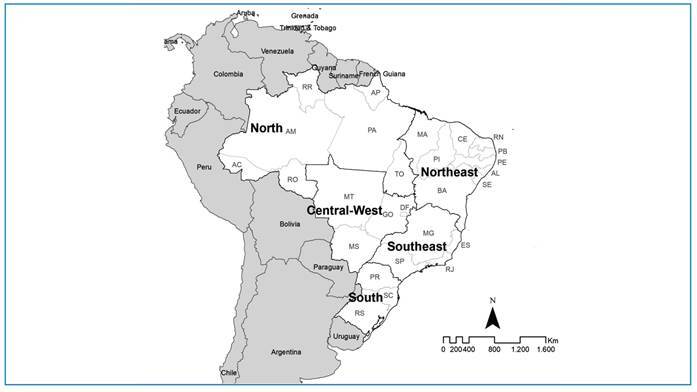
Study areas: states and regions of Brazil.

Geographically, Brazil is divided into five administrative regions (North, Northeast, Southeast, South, and Central-West), comprising 26 states and 1 federal district, with 5,570 municipalities serving as the analysis units (Figure 1). The country spans a territorial area of 8,510,417.771 km² and has approximately 203,080,756 inhabitants^11^.

North region (AC: Acre, AP: Amapá, AM: Amazonas, PA: Pará, RO: Rondônia, RR: Roraima, and TO: Tocantins), Northeast region (AL: Alagoas, BA: Bahia, CE: Ceará, MA: Maranhão, PB: Paraíba, PE: Pernambuco, PI: Piauí, RN: Rio Grande do Norte, and SE: Sergipe), Southeast region (ES: Espírito Santo, MG: Minas Gerais, RJ: Rio de Janeiro, and SP: São Paulo), South region (PR: Paraná, RS: Rio Grande do Sul, and SC: Santa Catarina), and Central-West region (DF: Federal District, GO: Goiás, MT: Mato Grosso, and MS: Mato Grosso do Sul).

Data regarding trachoma-associated HA (trachoma being the primary or secondary cause) and DC (trachoma being the underlying or associated cause) were extracted from the SIH and SIM databases, respectively, provided by the Department of Information Technology of the Unified Health System (in Portuguese: *Departamento de Informática do SUS*), Ministry of Health. The following International Classification of Diseases codes (in Portuguese: *Classificação Internacional de Doenças e Problemas Relacionados à Saúde* [CID10]) were used to identify patients with trachoma-associated HA and DC: trachoma (A71), early phase of trachoma (A71.0), active phase of trachoma (A71.1), unspecified trachoma (A71.9), and trachoma sequelae (B94.0).

The analysis involved calculating absolute and relative frequencies and crude and standardized average rates (per 10^6^ inhabitants) of the trachoma-associated HA and DC across various sociodemographic variables. The sociodemographic variables included sex (male or female), age group (0-14, 15-29, 30-39, 40-49, 50-59, 60-69, or ≥70 years), area of residence (capital or interior), ethnicity (white, black, brown, yellow, or indigenous), regions (North, Northeast, Central-West, South, or Southeast), population size of the municipality (small size I: ≤20,000 inhabitants; small size II: 20,001−50,000 inhabitants; medium size: 50,001−100,000 inhabitants; large size: >100,001 inhabitants), type of municipality according to the National Health Survey, and the Brazilian Deprivation Index (in Portuguese: *Índice Brasileiro de Privação* [IBP]) with reference to 2010 (very low, low, medium, high, or very high).

For the spatial distribution analysis, the average rates for the 2000-2004, 2005-2009, 2010-2014, 2015-2019, and 2020-2022 periods were calculated and standardized using the direct method based on the age structure by sex from the 2010 census (per 10^6^ inhabitants). The natural break method of the Jenks classification algorithm (*natural breaks*) categorized the spatial classes of the adjusted rates. The areas of residence for HA and DC were utilized as the units of analysis (26 states and 1 federal district), excluding patients from an unknown state of residence.

Statistical analyses were conducted using *Stata® version 11.2 software* (StataCorp, College Station, Texas), while *qGis® version* 2.18.6 (QGIS Geographic Information System. QGIS Association. http://www.qgis.org) facilitated spatial analysis and thematic mapping. 

### ● Ethical considerations

This study was approved by the Research Ethics Committee of the Hospital São José of Infectious Diseases of the Health Department of the State of Ceará (Approval number: 5.132.182).

## RESULTS

During 2000-2022 in Brazil, 141/263,292,807 HA related to trachoma were identified, with an adjusted mean rate of 0.031 per 10^6^ inhabitants. Additionally, 126/27,596,830 trachoma-related DC were recorded. Of the HA cases, 83.0% were primarily associated with trachoma, of which 12.1% progressed to death. Among the recorded DC, 8.7% cited trachoma as the underlying cause, and 83.3% of deaths occurred in hospital settings. Trachoma sequelae (CID10 code B94.0) were noted in 8.5% of HA and 6.3% of DC (Table 1, Table 2).

**TABLE 1:** Trachoma-related hospital admissions according to sociodemographic variables in Brazil (2000-2022).

Variables	Hospital admissionsª			
	N	%	Crude rate per 10^6^ inhabitants	Adjusted rate per 10^6^ inhabitants (95% confidence interval)
Total	141	100.0	0.031	0.031 (0.026-0.036)
**Trachoma as cause of hospital admission**				
Primary	117	83.0	-	-
Secondary	26	18.4	-	-
**Death during hospital admission**				
No	124	87.9	-	-
Yes	17	12.1	-	-
**Trachoma sequelae** ^c^				
Yes	12	8.5	-	-
No	129	91.5	-	-
**Sex**				
Female	63	44.7	0.028	0.027 (0.021-0.034)
Male	78	55.3	0.036	0.035 (0.028-0.043)
**Age group (years)**				
0-14	55	39.0	0.050	0.050 (0.037-0.063)
15-29	17	12.1	0.014	0.014 (0.008-0.021)
30-39	5	3.5	0.007	0.007 (0.001-0.013)
40-49	12	8.5	0.021	0.021 (0.009-0.032)
50-59	9	6.4	0.021	0.021 (0.007-0.034)
60-69	16	11.3	0.060	0.056 (0.029-0.084)
≥70	27	19.1	0.125	0.119 (0.074-0.163)
**Ethnicity**				
White	39	27.7	0.019	-
Black	4	2.8	0.012	-
Brown	24	17.0	0.496	-
Yellow	3	2.1	0.002	-
Indigenous	2	1.4	0.106	-
No information	69	48.9	-	-
**Residence in the capital**				
No	110	78.0	0.032	0.032 (0.026-0.038)
Yes	31	22.0	0.029	0.029 (0.019-0.039)
**Population size of the municipality**				
Small size I	19	13.5	0.027	0.024 (0.013-0.035)
Small size II	29	20.6	0.041	0.038 (0.024-0.052)
Medium size	14	9.9	0.027	0.026 (0.012-0.039)
Large size	79	56.0	0.032	0.032 (0.025-0.039)
**Brazilian Deprivation Index (2010)**				
Very low	20	14.2	0.025	0.023 (0.013-0.033)
Low	30	21.3	0.037	0.036 (0.023-0.049)
Medium	28	19.9	0.032	0.032 (0.020-0.043)
High	28	19.9	0.032	0.031 (0.020-0.043)
Very high	35	24.8	0.034	0.031 (0.020-0.041)
**Region**				
North	20	14.2	0.054	0.049 (0.026-0.071)
Northeast	33	23.4	0.026	0.026 (0.017-0.035)
Southeast	58	41.1	0.031	0.029 (0.022-0.037)
South	18	12.8	0.028	0.029 (0.015-0.042)
Central-West	12	8.5	0.036	0.038 (0.016-0.060)

Source: SIH

International Classification of Diseases codes (In Portuguese: *Classificação Internacional de Doenças e Problemas Relacionados à Saúde* [CID 10]) A71, A71.0, A71.1, A71.9, and B94.0] identified in the Hospital Admission Authorization (In Portuguese: *Autorização de Internação Hospitalar*)^a^ and death certificates^b^. ^c^Trachoma sequelae only using CID10 code B94.0.

**TABLE 2:** Trachoma-related death certificates according to sociodemographic variables in Brazil (2000-2022).

Variables	Death certificates^b^			
	N	%	Crude rate per 10^6^ inhabitants	Adjusted rate per 10^6^ inhabitants (95% confidence interval)
**Total**	126	100.0	0.028	0.028 (0.023-0.033)
**Trachoma as cause of death**				
Underlying	11	8.7	-	-
Associated	115	91.3	-	-
**Place of death**				
Hospital	105	83.3	-	-
Other healthcare establishments	6	4.8	-	-
Residence	14	11.1	-	-
Public highway	1	0.8	-	-
No information	0	0.0	-	-
**Trachoma sequelae** ^c^				
Yes	8	8 6.3	-	-
No	118	93.7		-
**Sex**				
Female	48	38.1	0.021	0.021 (0.015-0.027)
Male	78	61.9	0.036	0.035 (0.028-0.043)
**Age group (years)**				
0-14	7	5.6	0.006	0.006 (0.002-0.011)
15-29	7	5.6	0.006	0.006 (0.002-0.010)
30-39	5	4.0	0.007	0.007 (0.001-0.013)
40-49	13	10.3	0.022	0.022 (0.010-0.035)
50-59	10	7.9	0.023	0.023 (0.009-0.037)
60-69	21	16.7	0.079	0.074 (0.042-0.105)
≥70	63	50.0	0.292	0.277 (0.209-0.345)
**Ethnicity**				
White	53	42.1	0.025	-
Black	9	7.1	0.027	-
Brown	58	46.0	1.198	-
Yellow	0	0.0	0.000	-
Indigenous	0	0.0	0.000	-
No information	6	4.8	-	-
**Residence in the capital**				
No	95	75.4	0.028	0.028 (0.022-0.033)
Yes	31	24.6	0.029	0.029 (0.019-0.039)
**Population size of the municipality**				
Small size I	22	17.5	0.031	0.027 (0.016-0.039)
Small size II	29	23.0	0.041	0.040 (0.025-0.054)
Medium size	18	14.3	0.035	0.035 (0.019-0.051)
Large size	57	45.2	0.023	0.023 (0.017-0.030)
**Brazilian Deprivation Index (2010)**				
Very low	20	15.9	0.025	0.021 (0.012-0.030)
Low	22	17.5	0.027	0.026 (0.015-0.037)
Medium	20	15.9	0.023	0.023 (0.013-0.033)
High	29	23.0	0.033	0.034 (0.022-0.046)
Very high	35	27.8	0.034	0.035 (0.023-0.046)
**Region**				
North	8	6.3	0.021	0.030 (0.009-0.051)
Northeast	49	38.9	0.039	0.041 (0.029-0.052)
Southeast	45	35.7	0.024	0.022 (0.016-0.029)
South	18	14.3	0.028	0.025 (0.014-0.037)
Central-West	6	4.8	0.018	0.02 (0.004-0.036)

Source: SIM

International Classification of Diseases codes (In Portuguese: *Classificação Internacional de Doenças e Problemas Relacionados à Saúde* [CID 10]) A71, A71.0, A71.1, A71.9, and B94.0] identified in the Hospital Admission Authorization (In Portuguese: *Autorização de Internação Hospitalar*) ^a^ and death certificates^b^. ^c^Trachoma sequelae only using CID10 code B94.0.

**FIGURE 2:**
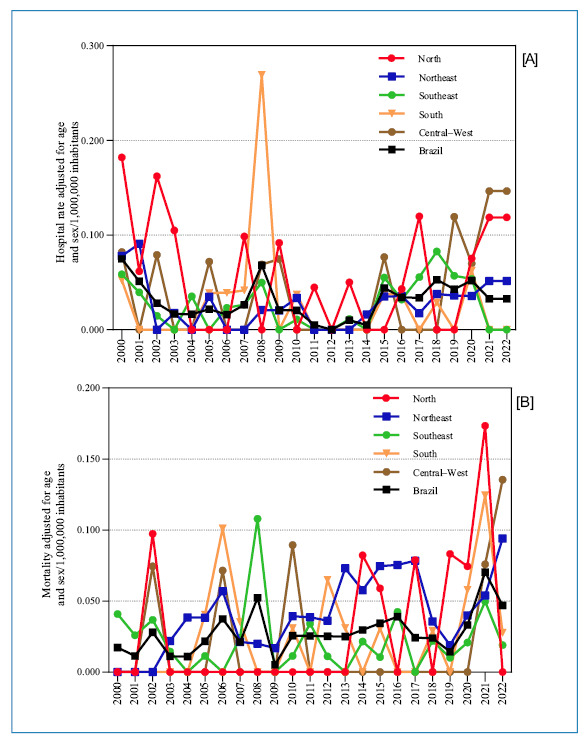
Trachoma-related **(A)** hospital admission rate and **(B)** mortality rate adjusted by sex and age in Brazil overall and each region (2000-2022).

The age- and sex-adjusted rates (per 10^6^ inhabitants) for HA and DC did not exhibit a discernible temporal pattern across Brazil and its regions throughout the study period (Figures 2A-2B, Table 1, Table 2).

Most of the HA comprised males (55.3%), individuals aged 0-14 years (39.0%), and those identifying as white (27.7%). The highest adjusted mean rates were observed in males, individuals aged ≥70 years, and those of mixed race (crude rate, 0.496/10^6^ inhabitants) (Table 1). Most DC were recorded for males (61.9%), individuals aged ≥70 years (50.0%), and those identifying as brown (46.0%). The highest adjusted average rates were observed in males, individuals aged ≥70 years and those identifying as brown (crude rate, 1.198/10^6^ inhabitants) (Table 2).

Regarding the municipality classification variables, trachoma-related HA were more frequent among residents of inland municipalities (78.0%), large size municipalities (56.0%), and areas with a very high IBP (24.8%). The highest adjusted rates per 10^6^ inhabitants were observed in residents of inland municipalities, small size II municipalities, and areas with a low IBP. Trachoma-related DC were more frequently recorded for residents of inland municipalities (75.4%), municipalities with >100,000 inhabitants (45.2%), and areas with a very high IBP (27.8%). The highest adjusted rates per 10^6^ inhabitants were observed in residents of capital cities, small municipalities with a population of 20,001−50,000 inhabitants, and areas with a very high IBP (Table 1).

Among the regions, trachoma-related HA were the most common in the Southeast (41.1%) and high adjusted average rates observed in the North region (Table 1). Trachoma-related DC and the highest adjusted average rates were observed in the Northeast region (Table 2).

**FIGURE 3:**
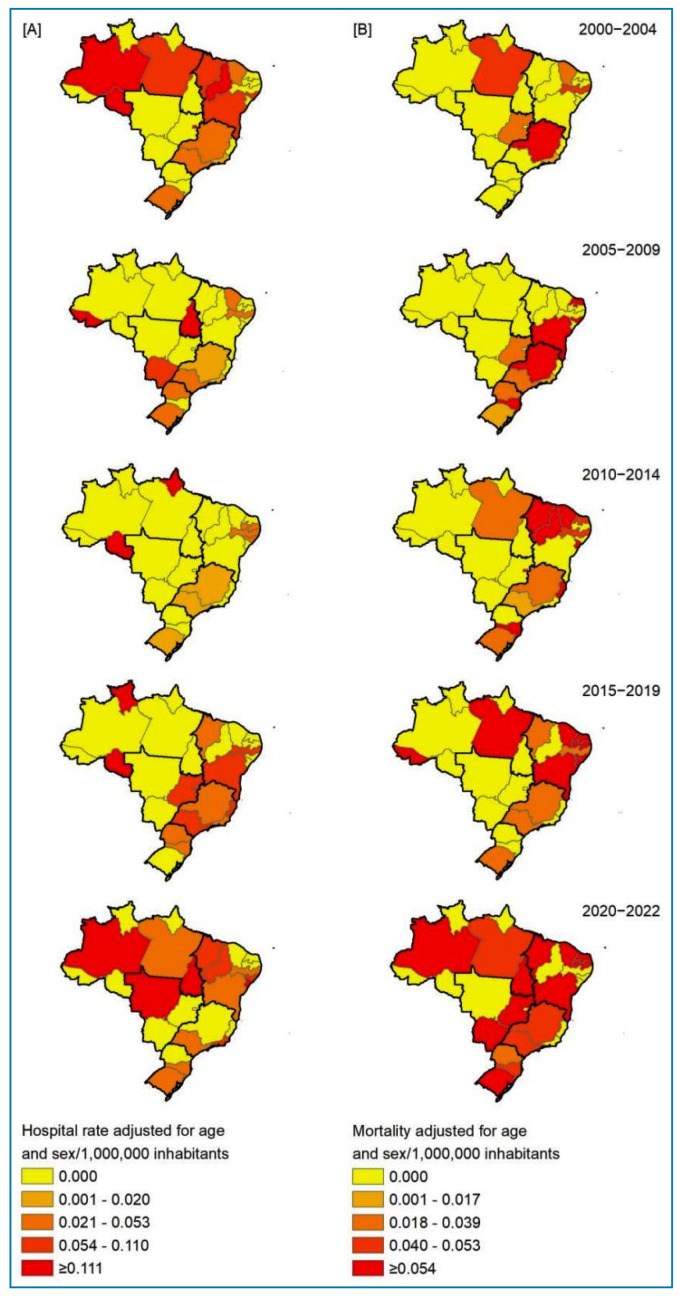
Spatial distribution by state of the trachoma-related **(A)** hospital admission rate and **(B)** mortality rate in Brazil (2000-2022).

The spatial distribution of trachoma-related HA and DC rates exhibits heterogeneity over time across the analyzed periods, with various states recording high rates (HA: ≥0.111 per 10^6^ inhabitants; DC: ≥0.054 per 10^6^ inhabitants) (Figure 3). This spatiotemporal pattern, varying across regions over the years (average adjusted rates every 5 years), is also evident in the annual time-series analysis of the country’s regions (Figures 2 and 3).

## DISCUSSION

Despite having identified the limitations, trachoma-related morbidity and mortality persist in Brazil. The spatial distribution of trachoma-related HA and deaths across the country indicates occurrences in all states, particularly in large municipalities and areas characterized by greater social inequality and vulnerability. The Northeast region of Brazil had the highest proportion of trachoma-related deaths, suggesting more than just an operational issue with registration. Notably, one state situated in the North region of Brazil, exhibited the highest trachoma-related mortality rate. Moreover, the North and Northeast regions had the lowest proportions of dwellings with access to a general water supply network (58.8% and 80.0%, respectively) and garbage collection (72.4% and 70.8%, respectively) in Brazil^11^. Hence, it is imperative to analyze these occurrences in areas where a significant portion of the Brazilian population faces limited access to healthcare services and resides in precarious living conditions^12^.

This study underscores the necessity of enhanced evaluation and monitoring of data within the country’s Health Information Systems (In Portuguese: *Sistemas de Informação em Saúde* [SIS]) to ensure higher quality of analysis. Although death is not a direct clinical outcome of trachoma, HA could be associated with the need for surgical correction of eyelid sequelae of the disease^5^. These occurrences may indicate more severe disease states and potential limitations in accessing timely care with greater technical complexity within the SUS^5^. A review of the registration of trachoma-related deaths and HA in a long-term national historical series further underscores these perspectives^9^.

Trachoma as a cause of death suggests a likely inconsistency in the registry, as it is directly associated with disability but not with death. In addition to this inference not correlating with the clinical status of the disease, inadequate coding of records with the clinical forms of trachoma, fragmentation and/or duplication, operational limitations and a lack of interoperability highlight the limited reliability of the records. This could lead to misinterpretation, making them unrepresentative of the population and/or the health-disease process in question^13^, and thus influencing the correct decision making for SUS management^14^.

A better understanding of the possible operational factors that may have influenced these results is warranted to bring positive changes in the process of management and analysis of health data from the SIH-SUS and SIM, especially considering the low endemicity of the disease in the country^7^ ^,^ ^8^ ^,^ ^9^. 

The difficulties experienced by the municipal management and local health professionals in conducting care and surveillance actions indicate the need for operational and implementation research related to the SIS to improve its use and enhance transparency in the database analysis strategies within the SUS^15^. Therefore, a detailed evaluation of the quality analysis reports of these systems is recommended to detect causes incompatible with the occurrence of these events, thereby improving the data adequacy and accuracy. This is crucial for planning public health policies aimed at eliminating trachoma.

The lack of qualified data for evidence-based decision-making^16^ and operational difficulties in managing and recording TT cases^17^ ^,^ ^18^ in information systems make it difficult to understand the trachoma morbidity and mortality patterns and eliminate the disease.

Underreporting and absence and/or inconsistency of information can lead to the under- or overestimation of health indicators. Therefore, the data collected by the services should be evaluated, and the country's health professionals should be trained to enhance the epidemiological quality of actions that closely align with the real situation^10^.

Nevertheless, the morbidity of the disease is considered relevant in terms of public health, with cases recorded in more than 9% of Brazilian municipalities (508) and associated with leprosy, leishmaniasis, and schistosomiasis in almost all (96.6%) cases detected as NTD in Brazil in 2015^10^. It is also worth considering the limited understanding of the record of hospitalizations due to the disease in the country, an understudied aspect, and the possible indirect impact of the disease on mortality^10^.

Global NTD programs acknowledge the strategic importance of progress in national health systems for more effective and planned responses to achieve the elimination targets set by the WHO^13^.

Furthermore, healthcare and surveillance interventions can be enhanced by using better quality data^14^. Therefore, monitoring the completeness of DC, as recommended by the WHO, is considered a strategic and essential approach for the SUS to obtain consistent information on mortality for conducting assertive interventions^19^.

Improvement in the quality of SIM records was particularly evident after 2006, with a reduction in records of deaths due to undetermined causes. The most critical aspects of the SIH-SUS are related to coverage and completeness due to imprecision in the definition of the cause of hospitalization^20^.

Therefore, the SIS used to characterize morbidity and mortality must provide a specific functional perspective, with individualized analysis through its own critical reports, in addition to the ability to interoperate with different databases^21^ that include trachoma. By utilizing probabilistic or deterministic resources and tools, data linkages expand the scope for qualifying the study of these diseases and provide space to ensure the care of people with these conditions^22^.

Furthermore, the systematic use of integrated data at the local, regional, and national management levels is essential for achieving the Sustainable Development Goals (SDGs) of the 2030 Agenda^23^ ^,^ ^24^. Furthermore, it is crucial to assess the composition and systematically monitor health information systems to qualify information and support the verification of elimination in populations at risk of the disease^2^.

In Brazil, trachoma remains among the diseases targeted for elimination^4^ ^,^ ^25^; however, it was not included among the NTDs presented in the National Agenda of Priorities for Health Research, and therefore lacks funding, as defined in that proposal^24^ ^,^ ^26^. Despite presenting a considerable global burden of disease (measured in disability-adjusted life years), particularly because it is associated with a low and very low “Human Development Index” and lower “expected years of schooling” dimension, there is a probable decrease in detection due to better socioeconomic and educational development conditions in the population^27^.

In 2023, trachoma was included in the scope of the prioritized socially determined diseases by the Interministerial Committee for the Elimination of Tuberculosis and Other Socially Determined Diseases (In Portuguese: *Comitê Interministerial para a Eliminação da Tuberculose e de Outras Doenças Determinadas Socialmente*). As progress has been made, the unfolding of the State Policy - Healthy Brazil Program and expansion of inter-sectoral actions have been aligned with the 2030 Agenda to achieve the SDGs. This focus aims to eliminate and/or reduce public health problems such as trachoma that affect populations facing social inequality^24^ ^,^ ^28^.

However, challenges associated with improving these inter-sectoral actions include ensuring completeness and consistency of records, monitoring them, and evaluating actions and strategic analyses by integrating information systems. Nonetheless, there are prospects for expanding the planning and decision-making capacity to control the disease prevalence in the country^23^.

The limitations of this study include the use of secondary data from the SIH-SUS and SIM, which may result in incomplete recording of variables. The results indicate the presence of probable operational inconsistencies in the databases and the need to validate the information for the true characterization of this NTD.

Despite these limitations, the use of large databases from different SISs combined with specific critical analyses tailored to these systems and spatial and temporal distributions provides new perspectives for efficient and reliable situational analysis of trachoma morbidity and mortality in the country.

Trachoma continues to impose high morbidity and mortality burdens on the country. However, there is an increased need for the evaluation, monitoring, and systematic critical operational analysis of the SIS data to ensure the completeness and consistency of HA and DC records. Expanding research to understand the factors influencing these outcomes better implies changes in the SIH and SIM management processes. Appropriation of this information will provide knowledge for health management and planning, with a view toward more qualified and integrated interventions for healthcare and surveillance, particularly in primary healthcare, which is essential for controlling trachoma and NTDs in general.

Despite the relatively low rates of trachoma morbidity in Brazil, the associated mortality rates are of concern. The heterogeneous patterns of occurrence in the country in terms of population and territory reinforce the need to evaluate and monitor the available data, despite the low prevalence, in order to achieve and maintain the elimination targets in Brazil in the future. Therefore, there is a clear need to qualify disease surveillance, care, and control interventions.
